# Child Anxiety, Depression, and Post-traumatic Stress Disorder Following Orthopedic Trauma

**DOI:** 10.7759/cureus.42140

**Published:** 2023-07-19

**Authors:** Ahmed S Al Zomia, Mahdi Mofarah Alqarni, Abdullah A Alaskari, Abdullah Al Qaed, Abdulrhman M Alqarni, Abdulrahman M Muqbil, Daher M Alshehri, Lama A Lahiq, Mohammed A Alhifthi, Yazeed Alshahrani

**Affiliations:** 1 Department of Medicine and Surgery, King Khalid University, Abha, SAU; 2 Department of Pediatric Orthopedic, Abha Maternity and Children Hospital, Abha, SAU; 3 Department of Medicine and Surgery, Umm Al-Qura University, Al Qunfudhah, SAU

**Keywords:** saudi arabia, prevalence, post-traumatic stress disorder, anxiety, depression, childhood trauma

## Abstract

Background: Many children and adolescents are exposed to different types of trauma, e.g., abuse or various disasters. Trauma can cause severe and long-term impairment and consequences, the most studied of which are post-traumatic stress disorder (PTSD) and PTSD symptoms (PTSS). PTSD is highly prevalent in clinical practice (with a frequency of about 7%) and is a debilitating consequence of trauma.

Aim: The current study aimed to assess childhood injuries and their associated anxiety, depression, and post-traumatic stress disorder following orthopedic trauma.

Methods: A descriptive cross-sectional study was conducted, including all pediatric patients with trauma at Abha Maternity and Children Hospital in the Seer region of Saudi Arabia, as well as pediatric patients with trauma at Abha Maternity and Children Hospital during the period from January 1, 2021 to December 31, 2022. Data were collected from the children’s caregivers using a direct interview questionnaire to assess the children’s personal data, depression, anxiety, and post-traumatic stress disorder. Children’s trauma-related data were extracted from their medical records using a pre-structured data extraction sheet.

Results: A total of 100 children with trauma were included. Children ranged in age from eight to 12 years, with a mean age of 7.3 ± 3.4 years. In all, 67 (67.0%) children were males, and only 6 (6.0%) had chronic health problems. The vast majority of the children with trauma had a low-severity experience of depression and anxiety following trauma (97.1% for each), and only one child had a high-severity experience of depression and anxiety. In all, 5 (4.9%) children with trauma experienced clinically significant PTSD, and the vast majority of them showed a low likelihood of the disorder. Multiple fractures and undergoing surgery were significant predictors of developing PTSD (P < 0.05).

Conclusion: In conclusion, the current study revealed that bone trauma was frequent among children, mainly due to playing accidents. Also, a low prevalence of post-traumatic stress disorders and their mental consequences was estimated.

## Introduction

Injuries are one of the principal causes of childhood morbidity and mortality [1]. Childhood injuries are a significant public health issue in the United States. According to the Centers for Disease Control and Prevention (CDC), more than 7,000 children and teens aged 0-19 died because of unintentional injuries in 2019, which averages out to about 20 deaths per day [2]. The leading causes of unintentional child injury include motor vehicle crashes, suffocation, drowning, poisoning, fires, and falls [3]. The number of children dying from injury dropped by nearly 30% over the last decade, although unintentional injury still accounts for one-third of deaths in children and adolescents each year, primarily from motor vehicle crashes [4]. It is important to note that child injury is often preventable, and parents and caregivers can take measures to reduce the risk of injury in children. The Injury Prevention Program (TIPP) offers resources for pediatricians to educate parents on injury prevention measures for children from newborn through 12 years of age, including topics such as motor vehicles, firearms, bicycle crashes, drowning, poisoning, choking, burns, falls, and pedestrian hazards [5-7].

Mental disorders in childhood are among the risk factors for childhood injuries [8]. They are responsible for about 14% of the global burden of disease, with depression and anxiety as the leading causes of disability worldwide [9]. These conditions often develop in childhood and adolescence and, if left untreated, can negatively impact psychosocial function and add to the healthcare burden. Moreover, psychiatric comorbidities are common in depressive and anxiety disorders and often lead to increased symptom severity and an overall worse outcome. As such, early diagnosis and intervention are vital [10-13]. Our goal is to determine child anxiety, depression, obsessive-compulsive disorder, and post-traumatic stress disorder following orthopedic trauma in both male and female children across a range of age groups.

## Materials and methods

A descriptive cross-sectional study was conducted, including all pediatric patients with trauma at Abha Maternity and Children Hospital in the Seer region of Saudi Arabia, during the period from January 1, 2021 to December 31, 2022. Data were collected from children’s caregivers using a direct interview questionnaire to assess children’s depression, anxiety, and post-traumatic stress disorder using the RCADS-25, which stands for the Revised Children's Anxiety and Depression Scale-25. It is a questionnaire consisting of 25 items designed to gauge the presence and intensity of anxiety. This scale includes two subcategories: one for measuring anxiety levels and another for assessing depressive symptoms. Additionally, it provides an overall score. Both the anxiety and depression subscales demonstrate a strong correlation with clinically identified anxiety and depression groups. Each item in the scale evaluates the frequency of symptoms and is rated on a 4-point Likert scale. The questionnaire included the participant’s relationship to the child, demographic data, medical history, and other clinically relevant information. Also, children’s depression and anxiety were assessed using the Revised Children’s Anxiety and Depression Scale (RCADS-25) with two subscales (total anxiety and total depression) [14]. It can be used by children and adolescents between the ages of eight and 18. A parent (or caregiver) version is also available to rate a child's or adolescent’s level of anxiety and depressive symptoms based on personal observations. Post-traumatic stress disorder was assessed using the combined child trauma screen (CTS), which consists of four items for experienced events and six items for reactions [15]. Children’s trauma-related data were extracted from their medical records using a pre-structured data extraction sheet.

Data analysis

After the data were extracted, it was revised, coded, and fed to the statistical software IBM SPSS version 22 (SPSS, Inc., Chicago, IL). All statistical analysis was conducted using two-tailed tests. Differences were considered statistically significant at a P < 0.05. Childhood depression and anxiety were assessed by summing up all discrete item scores for each subscale (depression and anxiety). We gave the following scores for each question based on responses: 0 for never, 1 for once in a while, 2 for half the time, and 3 for almost always for how often the following things have bothered you in the last two weeks. Total scores are converted to T-scores using specific equations that have been developed through research and account for the gender and grade of each child. Converted scores on the subscales are divided into scoring ranges, where (a) scores below 65 represent low severity, (b) scores between 65 and 70 represent medium severity and are on the borderline clinical threshold, and (c) scores above 70 represent high severity and are above the clinical threshold [16]. As for PTSD, CTS scores for reaction items are summed up, and a total on the CTS of 6 or greater on the child report indicates a high likelihood that the child may be suffering from clinically significant levels of PTSD symptoms [15]. A descriptive analysis based on frequency and percent distribution was performed for all variables, including children’s personal data, medical history, trauma data, management, ICU, and duration of hospital admission. Also, students’ depression and anxiety levels, along with PTSD, were graphed. Crosstabulation was used to assess factors associated with PTSD among children using Pearson’s chi-square test and the exact probability test for small frequency distributions.

## Results

A total of 100 children with trauma were included. Data were collected from the fathers of 80 (80.0%) and the mothers of 20 (20.0%) children. The children’s ages ranged from 1 to 12 years, with a mean age of 7.3 ± 3.4 years. In all, 67 (67.0%) children were males, and only 6 (6.0%) had chronic health problems. As for children’s weight for age, 46 (46.0%) were underweight, 27 (27.0%) had a normal weight for age, and 27 (27.0%) were overweight or obese. Only 5 (5.0%) children had a family history of psychiatric disease (Table 1). 

**Table 1:** Personal characteristics of study children with trauma and respondents, Abha Maternity and Children Hospital, Saudi Arabia.

Personal data	No	%
Respondent		
Father	80	80.0%
Mother	20	20.0%
Child age in years		
5–9	45	55.0%
10–12	45	45.0%
Child gender		
Male	67	67.0%
Female	33	33.0%
Child had chronic disease		
Yes	6	6.0%
No	94	94.0%
Weight for age		
Underweight	46	46.0%
Normal	27	27.0%
Overweight	14	14.0%
Obese	13	13.0%
Family history of psychiatric diseases		
Yes	5	5.0%
No	95	95.0%

Children’s injury-related data, types, and sites, Abha Maternity and Children Hospital, Saudi Arabia (Table 2). The duration of trauma was less than six months in around one-fourth of the studied children (24.0%). Two-fifths of the children experienced trauma more than six months ago but less than one year (40.0%), and finally, nearly one-third of the children had trauma more than one year ago (36.0%). The most reported cause of injury was during play (44.0%) and fall from a height (37.0%). Trauma was at the upper extremities among 50 (50.0%), which was unilateral among 65 (97.1%) of them, and at the head among 31 (31.0%). Only 7 (22.6%) experienced loss of consciousness after head trauma. In all, 78 (78.0%) children had only one trauma, and 16 (16.0%) had two trauma injuries.

**Table 2:** Children’s injury-related data, types, and sites, Abha Maternity and Children Hospital, Saudi Arabia. RTA: road traffic accident.

Injury data	No	%
Type of injury		
Orthopedic injury	87	87.0%
Head injury	12	11.7%
Tissue injury	1	1.0%
Duration of trauma		
Less than six months	24	24.0%
6–12 Months	40	40.0%
More than one year	36	36.0%
Cause of trauma		
RTA	10	10.0%
Fall from height	37	37.0%
During playing	44	44.0%
Others	9	9.0%
Site of trauma		
Head	31	31.0%
Upper extremities	50	50.0%
Lower extremities	17	17.0%
Pelvis	2	2.0%
If in extremities, is it		
Unilateral	65	97.1%
Bilateral	2	2.9%
Loss of consciousness after head injury		
Yes	7	22.6%
No	24	77.4%
Number of fractures		
One	78	78.0%
Two	16	15.5%
Three	3	2.9%
More	3	2.9%

Clinical data and hospital stay among children with trauma, Abha Maternity and Children Hospital, Saudi Arabia (Table 3). A total of 70 (70.0%) children had undergone surgery for trauma, and 10 (10.0%) needed ICU admission. As for hospital stays, 38 (38.0%) children were hospitalized for one to two days, 21 (21.0%) for three to four days, and 30 (20.0%) for five to seven days, but only 11 (11.0%) were hospitalized for more than seven days.

**Table 3:** Clinical data and hospital stay among children with trauma, Abha Maternity and Children Hospital, Saudi Arabia.

Clinical data	No	%
ICU admission		
Yes	10	10.0%
No	90	90.0%
Undergone surgery		
Yes	70	70.0%
No	30	30.0%
Days of hospital admission		
1–2 Days	38	38.0%
3–4 Days	21	21.0%
5–7 Days	30	30.0%
>7 Days	11	11.0%

**Figure 1:**
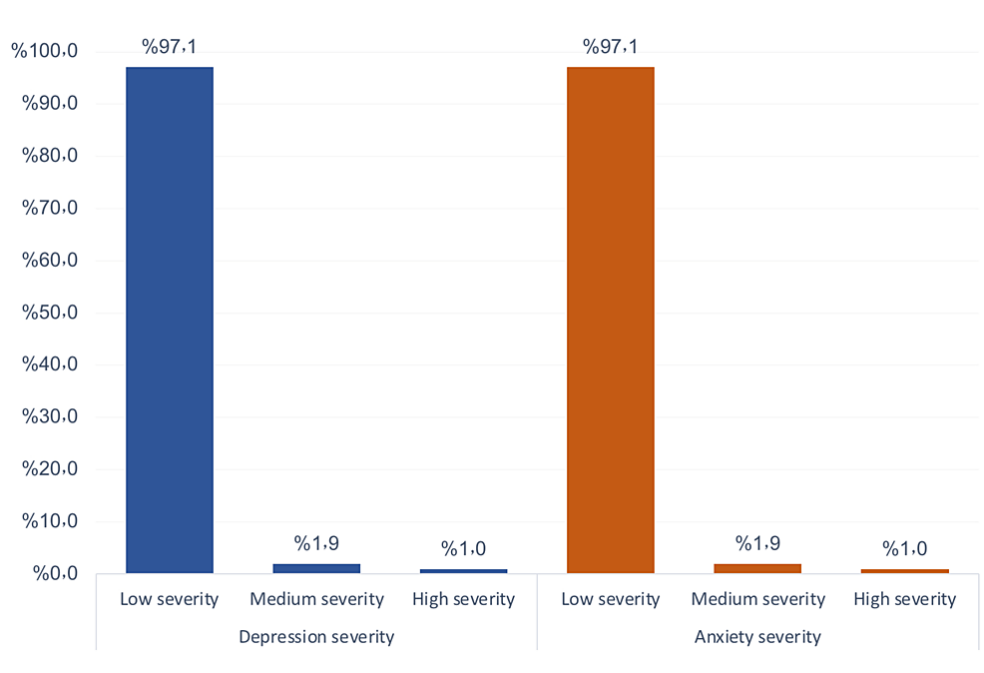
Prevalence of depression and anxiety among children following trauma in Abha Maternity and Children Hospital, Saudi Arabia.

Prevalence of depression and anxiety among children following trauma in Abha Maternity and Children Hospital, Saudi Arabia (Figure 1). The vast majority of the children with trauma had a low-severity experience of depression and anxiety following trauma (97.1% for each), and only one child had a high-severity experience of depression and anxiety.

Combined child trauma screening assessment among children in Abha Maternity and Children Hospital, Saudi Arabia (Table 4). Regarding experienced events, 15.0% of the children had seen people pushing, hitting, or throwing things at each other or stabbing, shooting, or trying to hurt each other; 10.0% experienced very upsetting or scary events; and 2.0% were hurt, punched, or kicked by someone with hands, belts, or other objects or tried to shoot or stab them. With regard to children’s reactions to events, 21.0% had trouble concentrating or paying attention, 18.0% of the children had sleep troubles, 16.5% tried to stay away from people, places, or things that reminded them of something that had happened, and 13.0% felt alone and not close to people around them.

**Table 4:** Combined child trauma screening assessment among children in Abha Maternity and Children Hospital, Saudi Arabia. PTSD: post-traumatic stress disorder.

PTSD	Yes			No				
Events frequency	Number		%	Number		%		
Has your child ever seen people pushing, hitting, or throwing things at each other or stabbing, shooting, or trying to hurt each other?	15		15.0	85.0		85.0		
Has someone ever really hurt your child, i.e., hit, punched, or kicked them with hands, belts, or other objects or tried to shoot or stab them?	2		2.0	98.0		98.0		
Has someone ever touched your child on the parts of their body that a bathing suit covers, in a way that made you or your child uncomfortable? Has someone had your child touch them in that way?	0		0.0	100		100.0		
Has anything else very upsetting or scary happened to your child?	10		10.0	90		90.0		
Reactions to experienced events	Never/rarely		One to two times per month		One to two times per week		Three+ times per week	
	No	%	No	%	No	%	No	%
Your child has strong feelings in their body when they remember something that happened (sweating, rapid heartbeat, feeling sick).	90	90.0	9	9.0	0	0.0	1	1.0
Your child tries to stay away from people, places, or things that remind them of something that happened.	83	83.0	13	13.0	3	3.0	1	1.0
Your child has trouble feeling happy.	90	90.0	7	7.0	1	1.0	2	2.0
Your child has trouble sleeping.	82	82.0	15	15.0	1	1.0	2	2.0
It is difficult for your child to concentrate or pay attention	79	79.0	13	13.0	5	5.0	3	3.0
Your child feels alone and not close to people around them.	87	87.0	11	11.0	0	0.0	2	2.0

**Figure 2:**
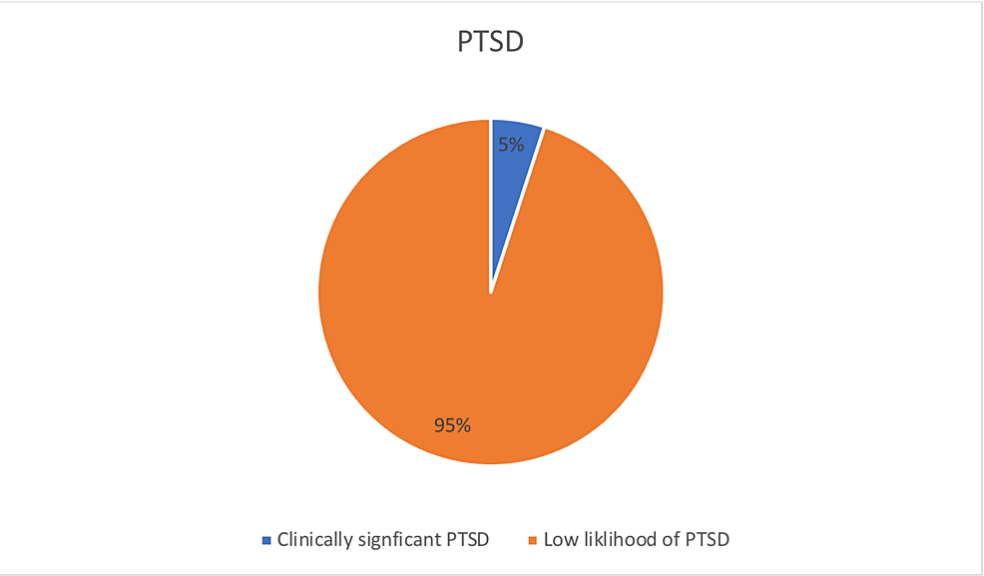
Post-traumatic stress disorder among children in Abha Maternity and Children Hospital, Saudi Arabia. PTSD: post-traumatic stress disorder.

Post-traumatic stress disorder among children in Abha Maternity and Children Hospital, Saudi Arabia (Figure 2). In all, 5 (5.0%) children with trauma experienced clinically significant PTSD, and the vast majority of them showed a low likelihood of the disorder.

Factors associated with PTSD among children in Abha Maternity and Children Hospital, Saudi Arabia (Table 5). PTSD was detected among 33.3% of children with three or more fractures versus 3.8% of others with less with recorded statistical significance (P = 0.009). Also, 7.4% of children who underwent surgery had PTSD compared to none of the others who did not (P = 0.049).

**Table 5:** Factors associated with PTSD among children in Abha Maternity and Children Hospital, Saudi Arabia. PTSD: post-traumatic stress disorder. P: Exact probability test. *P < 0.05 (significant).

Factors	PTSD				p-value
	Low likelihood of PTSD		Clinically significant PTSD		
	No	%	No	%	
Child’s age					0.347
5–9	40	95.2%	2	4.8%	
10–12	25	89.3%	3	10.7%	
Child gender					0.808
Male	61	95.3%	3	4.7%	
Female	34	94.4%	2	5.6%	
Child had chronic disease					0.165
Yes	5	83.3%	1	16.7%	
No	90	95.7%	4	4.3%	
Family history of psychiatric diseases					0.605
Yes	5	100.0%	0	0.0%	
No	93	94.7%	5	5.3%	
Duration of trauma					0.422
Less than six months	24	100.0%	0	0.0%	
6–12 Months	39	92.9%	3	7.1%	
More than one year	35	94.6%	2	5.4%	
Site of trauma					0.456
Head	31	100.0%	0	0.0%	
Upper extremities	45	91.8%	4	8.7%	
Lower extremities	17	94.4%	1	5.6%	
Pelvis	2	100.0%	0	0.0%	
Number of fractures					0.009*
One	76	96.2%	3	3.8%	
Two	15	100.0%	0	0.0%	
Three	2	66.7%	1	33.3%	
More	2	66.7%	1	33.3%	
ICU admission					0.426
Yes	8	88.9%	1	10.0%	
No	87	95.6%	4	4.4%	
Undergone surgery					0.049*
Yes	63	92.6%	5	7.4%	
No	32	100.0%	0	0.0%	
Days of hospital admission					0.623
1–2 Days	36	94.7%	2	5.3%	
3–4 Days	22	100.0%	0	0.0%	
5–7 Days	27	93.1%	2	6.9%	
>7 Days	10	90.9%	1	9.1%	

## Discussion

Childhood injuries can have a significant impact on an individual's future. From broken bones to head injuries, these experiences can stay with someone for years to come [17]. It is important to address these injuries as soon as they occur, both for physical healing and for future psychological well-being [18]. Continuing to struggle with the physical and emotional scars caused by such experiences can lead to a variety of psychological problems. Some common problems might include depression and anxiety, as well as post-traumatic stress disorder [19]. Those who have experienced childhood injuries may also struggle with trust issues and problems forming attachments with others later in life [20]. It is important for individuals who have suffered childhood injuries to seek professional help when necessary, in order to fully heal and move forward in a healthy way.

The current study aimed to assess childhood injuries, with their associated anxiety, depression, and post-traumatic stress disorder following orthopedic trauma. The study revealed that half of the children had upper extremity fractures, and about one-third had head trauma, which was mainly sustained during children’s playtime and falls from a height. The vast majority of children had one fracture, but some children had more than one. Similar findings were reported by Dave et al. [21], as fall-related injuries had the highest prevalence (3.38%), followed by road traffic injuries (RTI) (1.62%). In Saudi Arabia, Albedewi et al. [22] reported that falls represented 31.9% of childhood injuries, while 25.1% were due to motor vehicle collisions (MVC). The leading cause of fractures was falls (37.9%), followed by MVC (21.5%). The leading cause of burns was flames (52.1%), followed by scalding (36.4%). Another study revealed that the main cause of injury mortality was RTA (60.6%), which was most commonly found among 13-18-year-olds, followed by drowning at 13.4% and most commonly found among 6-12-year-olds [23].

Regarding injury-associated psychological disorders, the current study showed that most of the children with trauma had a low-severity experience of depression and anxiety following trauma, but only one child had a high-severity experience of depression and anxiety. Also, very few children with trauma experienced clinically significant PTSD, while the vast majority of them showed a low likelihood of the disorder. A study revealed that about 60% of children and adolescents have been exposed to potentially traumatic events (PTEs). Of these exposed children, approximately 30% afterward develop clinically significant PTSD; most will only experience ephemeral symptoms, whereas a few unfortunate individuals will experience more chronic life-long sequelae [24,25]. Recent estimates propose that 10% of children younger than 18 years of age are diagnosed with PTSD, and girls are four times more likely than boys to develop it [26]. Al-Saadi et al. [27] conducted a systematic review and found that the pooled prevalence rates of anxiety, depression, and PTSD among children were 13.9% (n = 1971; 95% confidence interval (CI) = 10.23-18.07%), 20.3% (n = 1990, 95% CI = 13.85-27.93%), and 20.0% (n = 755, 95% CI = 13.28-29.73%), respectively. Kolaitis [12] estimated that of those children and adolescents exposed to trauma, about 16% will develop PTSD: almost 10% as a consequence of non-interpersonal traumatic events and 25% following interpersonal traumas. Much higher rates were detected in Uganda, where 60% and 39% of participants fulfilled the diagnostic criteria for PTSD and depression, respectively [28]. The responses of children to trauma share broad similarities to those of traumatized adults, as well as some differences related to the plasticity of the developing brain and the child’s imaginative responses to the trauma of others. Post-traumatic stress disorders are viewed as the maladaptive persistence of a previously adaptive set of mental and physiologic responses to the trauma, organized as malignant memories [29].

## Conclusions

In summary, our study found that bone injuries were common in children, often resulting from accidents during play or falls from heights. However, the majority of these injuries were not severe or multiple, with only a small percentage requiring surgical intervention or admission to the intensive care unit (ICU). This finding helps to explain the relatively low prevalence of post-traumatic stress disorders and their associated mental consequences in this population. It is recommended to conduct longitudinal large-scale studies in order to gain a comprehensive understanding of the long-term effects and outcomes in younger patients. Additionally, it is important to modify psychotherapies to better suit the specific needs of these patients. By tailoring therapeutic approaches to address the unique challenges and developmental considerations of younger individuals, we can provide more effective and targeted interventions for their mental well-being.
